# Management and outcome variability in hernia-related small bowel obstruction: insights from the SnapSBO study

**DOI:** 10.1093/bjsopen/zraf127

**Published:** 2025-11-24

**Authors:** Matteo Maria Cimino, Gary Alan Bass, Hayato Kurihara, Gabriele Bellio, Matteo Porta, Luigi Cayre, Shahin Mohseni, Matthew J Lee, Lewis J Kaplan, Isidro Martinez-Casas, ESTES SnapSBO Group, Matteo Maria Cimino, Gary Alan Bass, Isidro Martinez-Casas, Lewis J Kaplan, Hayato Kurihara, Matthew J Lee, Shahin Mohseni, Pavel Karasek, Agron Dogjani, Kastriot Subashi, Klevis Doci, Joana Spaho, Abdulla, Sara Ahmed, Yusuf AlAnsari, Mariam AlKooheji, Alaa Marzooq, Khaled Nazzal, Emir Ahmetašević, Zlatan Mehmedović, Maja Kovačević, Jasminka Mujkanović, Peter Svenningsen, Marie Peter Møller, Gitte Emilje Olsen, Abeer Aboalazayem, Muhammad Ashrad Awad, Mahmoud M A Elfiky, Moemen Farouk, Mostafa Gad, Basma Magdy, Peep Talving, Edgar Lipping, Edgar Lipping, Sten Saar, Artjom Bahhir, Maarja Talviste, Vincent Dubuisson, Luca Cigagna, Spyridon Christodoulou, Panagiotis Kokoropoulos, Ioannis Margaris, Maria Papadoliopoulou, Theodoros A Sidiropoulos, Panteleimon Vassiliu, Evangelos Barkolias, Pavlos Georgalis, Theodosios Kantas, Vasiliki Nikolaou, Aristeidis Papadopoulos, Katerina Tata, Stergios Arapoglou, Ioannis Gkoutziotis, Aikaterini Mpratko, Elissavet Symeonidou, Stylianos Kykalos, Nikolaos Machairas, Adam Mylonakis, Panagiotis Sakarellos, Dimitrios Schizas, Michail Vailas, Iraklis Anastasiadis, Parmenion Patias, Koumarelas Konstantinos, Mourtarakos Saradis, Charles Lee, Chloe Spillane, Dylan Viani Walsh, Nadia Walsh, Arnold D K Hill, Thomas Noel Walsh, Gabriel Orsi, Andrew Keane, David Kearney, Emma de Sousa, Michael Sugrue, Anne Marie Doyle, Robert Fitzsimmons, Angus J Lloyd, Mohammad Saad Qasim, Mashood Ahmed, Taylor Jacoby, Michael E Kelly, Shafagh Khodadi, Paul McCormick, Éanna J Ryan, Mahmoud M Salama, Helen Heneghan, Cian Davis, Odhran K Ryan, Sean T Martin, Miklosh Bala, Michele Altomare, Stefano P B Cioffi, Andrea Spota, Giada Panagini, Laura Benuzzi, Stefania Cimbanassi, Noemi DiFuccia, Stefano Manfroni, Alan Biloslavo, Paola Germani, Nicolo de Manzini, Manuela Mastronardi, Anna Modica, Serena Scomersi, Gabriele Bellio, Luigi Cayre, Gaia Altieri, Pietro Fransvea, Gabriele Sganga, Silvia Tedesco, Francesca Bunino, Sabrina Caspani, Daniele DelFabbro, Simone Giudici, Giulia Mauri, Paolo Meneghesso, Enrico Ortolano, Carolina Perali, Antonella D'addiego, Francesca Di Vittorio, Gabriele Bormolini, Michele Carlucci, Alessia Malagnino, Maria Ilaria Ficaccio, Giovanni Pesenti, Claudia Tintori, Mauro Zago, Martina Zambon, Simona Meneghini, Andrea Mingoli, Giulia Duranti, Gioia Brachini, Pierfrancesco Lapolla, Mehdi Hanafi, Clara Valdez Cruz, Andrea Alfredo Huerta de León, Jose García Regalado, Pasquale de Jesús Cristiano Nakhal, Diego Enrique Rodríguez González, Jose Alaniz-Ruiz, Edgard Lozada-Hernández, Salvador Carlos-Jiménez, Oscar Reyes-Delgado, Monserrat Martínez-Zamorano, Ademola Adetoyese Adeyeye, Ehis Afeikhena, Akinola Akinmade, Babatunde Mustapha, Jaroslav Presl, Patrick Rebnegger, Bjoern Rudisch, Gruenfelder Johanna, Rokitte Karin, Filipa M CorteReal, Jorge A Pereira, Joao L Pinheiro, Daniela M Pinto, Andreia J Santos, Andreia M Silva, Susana Henriques, Joao Melo, António Miguel Pereira, Antonio Miguel Pereira, Ana Margarida Cabral, Bruno Dias Couto, Barbara Nunes Gama, Catarina Santos Rodrigues, Mara Nunes, Bruno Ribeiro Silva, Daniela Tavares, Toma Mihai, Oprea C Valentin, Srdjan S Putnik, Petar Andjic, Marija Djujic, Rastislav Filko, Vanja Kunkin, Andjela Milak, Aleksandar Ognjenovic, Nebojša Mitrović, Dejan Stevanović, Damir Jašarović, Goran Aleksandrić, Nemanja Trifunović, Mihailo Bezmarević, Sasa Dragović, Milan Jovanović, Bosko Milev, Miroslav Mitrović, Srdjan Petković, Valentina Isakovic, Nikola Zoran Nikolic, Predrag Radic, Dragan Luka Vasic, Zlatibor M Loncar, Dusan D Micic, Vladimir R Resanovic, Pavle D Vladimir, Krstina S Doklestic Vasiljev, Zlatibor M Loncar, Dusan D Micic, Vladimir R Resanovic, Pavle D Gregoric, Velibor Ljiljana Milic, Vladica Velibor Cuk, Jovan Todor Juloski, Radisav Slavoljub Radulovic, Dragana Dragan Arbutina, Jacobo Trebol, Isaac Tapiador-López, Manuel J Torres-Jurado, Andres E Valera-Montiel, Francisco Blanco-Antona, Beatriz de Andrés-Asenjo, Maria Ruiz-Soriano, Tania Gómez-Sanz, Andrea Vázquez-Fernández, Juan Beltran de Heredia, Cristina Rey-Valcárcel, Monica Ballón-Bordon, Maria Pérez-Díaz, Maria Dolores Sanchez-Rodriguez, Jose David Gonzalez-Esteban, Celia Alegre Nevado, Ricardo Montenegro Romero, Inés Capitán del Río, Andrea Campos-Serra, Raquel Gracia-Roman, Heura Llaquet-Bayo, Anna Muñoz-Campaña, Giulia Vitiello, Lorena Apodaca Murguiondo, Inigo Augusto Ponce, Amaia Garcia Dominguez, Aintzane Lizarazu Perez, Elena Sagarra Cebolla, Mónica García Aparicio, Paloma Garaulet González, Benito Miguel Josa Martínez, Miriam Fraile Vasallo, Mónica Mengual Ballester, Isabel Andrés Lucas Zamorano, Jose Martinez Moreno, Manuel Luis Buitrago Ruiz, Clara Piñera Morcillo, Alberto Díaz García, Hanna Hernández Oaknin, Maria Pellicer Barreda, Jennifer Amparo García Niebla, Antonio Pérez Álvarez, Diego Cordova, Laura Jiménez, Fernando Mendoza, Cristina Vera, Alberto Vilar Tabanera, María de los Ángeles Gil-Olarte Márquez, José Antonio López-Ruiz, Mª Estela Romero-Vargas, Julio Reguera-Rosal, Alberto García-García, Beatriz Marenco de la Cuadra, Eduardo Perea del Pozo, Virginia Duran Muñoz, Felipe Pareja Ciuró, Ainoa Benavides dos Santos, Ernest Bombuy, Anna G-Monferrer, Sandra López Gordo, José Guerra, Vanessa Sojo, Begona De Soto, Aaron Roman, Ana María González-Castillo, Elena Manzo, Estela Membrilla-Fernandez, Amalia Pelegrina-Manzano, Simone Cremona, Alexander Forero-Torres, Santiago Valderrabano, Francisco Reinoso Olmedo, Fuad Lopez Fernandez, Aitor Landaluce-Olavarria, Jon Barrutia- Leonardo, Alba Garcia-Trancho, Melania Gonzalez-De Miguel, Izaskun Markinez-Gordobil, Maryam Makki, Dana Altamimi, Sadhika Vinod, Olga Rutka, John V Taylor, M Denton, S Gourgiotis, R Ravi, A J Ribbits, Jared Wohlgemut, Niroshini R Hemadasa, Shehryar Rangana Khan, Christopher Leiberman, Sabreen P Elbakri, Charlie A Edgar, Conor Magee, Kamalesh Inteti, Oluwaseun Oyekan, Mehwish Ansar, Jeremy Wilson, Rahel Rashid, Deborah Atwell, Joshua Cassedy, Brianna Gabriel, William Hoff, Shyam Murali, Anna E Garcia Whitlock, Therese Murphy, Carolyn Susman, Sarah Barnett, Emily Ertmann, Camden DeSanctis, Pavel Karasek, Nathan Klingensmith, Dale F Butler, Brandon Bruns, Ankeeta Mehta, Vanessa Nomellini, Keyur Patel, Anthony Tannous

**Affiliations:** Department of Emergency Surgery, Fondazione IRCCS Ca’ Granda Ospedale Maggiore Policlinico, Milan, Italy; Division of Traumatology, Surgical Critical Care and Emergency Surgery, Perelman School of Medicine, University of Pennsylvania, Philadelphia, Pennsylvania, USA; Department of Biostatistics, Epidemiology and Informatics, Perelman School of Medicine, University of Pennsylvania, Philadelphia, Pennsylvania, USA; Department of Emergency Surgery, Fondazione IRCCS Ca’ Granda Ospedale Maggiore Policlinico, Milan, Italy; Department of Emergency Surgery, Fondazione IRCCS Ca’ Granda Ospedale Maggiore Policlinico, Milan, Italy; Department of Emergency Surgery, Fondazione IRCCS Ca’ Granda Ospedale Maggiore Policlinico, Milan, Italy; Department of Emergency Surgery, Fondazione IRCCS Ca’ Granda Ospedale Maggiore Policlinico, Milan, Italy; School of Medical Sciences, Örebro University, Örebro, Sweden; Department of Applied Health Research, University of Birmingham, Birmingham, UK; Division of Traumatology, Surgical Critical Care and Emergency Surgery, Perelman School of Medicine, University of Pennsylvania, Philadelphia, Pennsylvania, USA; Trauma and Emergency Surgery Unit, Hospital Universitario Virgen del Rocio, Sevilla, Spain

**Keywords:** patient outcome assessment, evidence-based practice, prospective studies

## Abstract

**Background:**

Small bowel obstruction (SBO) due to hernia remains a prevalent surgical emergency disproportionately affecting elderly and co-morbid populations. Limited high-quality data exist to guide evidence-informed interventions for hernia-related SBO. This study explored the management and outcomes of hernia-related SBO (hSBO) for patients captured in European Society for Trauma and Emergency Surgery (ESTES) SnapSBO database.

**Methods:**

SnapSBO is a prospective multicentre time-bound study that accrued consecutive inpatient admissions between November 2023 and May 2024. The present analysis was restricted to patients with abdominal wall hernias. Management pathways were categorized as direct to surgery (DTS), successful non-operative management (NOM), or surgery after trial of NOM (NOM-T). Outcomes of interest included complications, 30-day in-hospital mortality, length of hospital stay (LOS), and functional recovery assessed through patient-reported outcome measures (PROMs) using the PRO-diGI tool.

**Results:**

Among 1737 patients, SBO due to abdominal wall hernia was noted in 386. The median patient age was 73 (range 16-98) years, with 64.8% of patients aged > 65 years. Primary inguinal/abdominal wall hernias were the most common (62.2%). Of the patients, 51.6% were categorized as DTS, where 17.1% required surgery after NOM-T. NOM was successful in 31.2% of patients. Parastomal hernia management led to the highest complication rate (57.1%) and prolonged postoperative LOS (mean(standard deviation) 9.1(4.8) days; *P* = 0.030) compared with other hernia types. Functional recovery measured in 218 patients was significantly worse in those with parastomal hernia than in those with incisional or primary inguinal hernias (mean(standard deviation) bowel function scores 68.6(22.5) *versus* 83.6(17.6) and 82.0(20.3), respectively; *P* = 0.009).

**Conclusion:**

There is significant variability in practice and outcomes for hSBO management. Patients with parastomal hernias represent a high-risk subgroup. Future research should focus on PROMs and in developing evidence-based, context-specific guidelines for hSBO management.

## Teaser

Patient-related outcome measures should be taken into consideration when managing small bowel obstruction (SBO). Initially, non-operative management may be used in patients with hernia-related SBO. SnapSBO identified patients with parastomal hernias as a high-risk subgroup, with significantly higher complication rates, prolonged hospital stays, and worse functional recovery metrics.

## Introduction

Small bowel obstruction (SBO), a common surgical emergency, stems from multiple aetiologies, including adhesions, internal herniation, and congenital or acquired abdominal wall defects^1^. Although other types of SBOs seem to share some common initial management approaches, the absence of standardized protocols for hernia-related SBO (hSBO) care contributes to significant practice and outcome variability. Such variability is expressed as disparate approaches to the timing and appropriateness of surgical *versus* non-operative management for hSBO. Acute presentation poses additional challenges, because decisions are often influenced by patient factors, including co-morbidities, frailty, malnutrition, and hernia defect complexity, particularly in subgroups such as those with parastomal hernia^2,3^. Furthermore, traditional metrics such as the length of hospital stay (LOS) or complication rates provide an incomplete assessment of recovery, with functional outcomes and patient-centred metrics remaining largely underexplored^4,5^. It is anticipated that a robust understanding of these dimensions will enable the development of actionable clinical strategies to improve the quality and consistency of care^4^.

Rather than examining clinical trial data, which would eliminate the variability characteristic of current practice, an environmental scan of delivered care more usefully explores outcomes. Such study design, known as a snapshot audit, hew to a well characterized methodology^6^. Accordingly, the aims of the present study were to delineate management patterns and outcomes in hSBO and to examine the influence of hernia aetiology. This study leverages data from the European Society for Trauma and Emergency Surgery (ESTES) SnapSBO multinational prospective observational cohort study to examine hSBO care and characterize how care approaches influence outcomes.

## Methods

### Study design and setting

The SnapSBO study examined the care and outcomes of patients with mechanical SBO regardless of aetiology. The present subgroup analysis specifically focuses on SBO due to abdominal wall hernias. The study protocol, which adhered to the STROBE guidelines^7^, was preregistered with ClinicalTrials.gov (NCT05843097). Centre participation was unrestricted by geographic or institutional characteristics. Data collection spanned 6 months (1 November 2023–31 May 2024), during which consecutive adult inpatient admissions for mechanical SBO (of any aetiology) were identified and included. In the present study, data relating to consecutive adults with radiologically confirmed SBO attributable to a primary, incisional, or parastomal abdominal wall hernia were analysed. Patients in whom obstruction arose solely from postoperative adhesions remote from a hernia were excluded.

Institutional review board/ethics approval was secured from the institutional review boards of all participating centres. Data management complied with the European Union General Data Protection Regulation (GDPR; EU 2016/679) and *Health Insurance Portability and Accountability Act* (United States of America, 1996).

### Outcomes of interest

Primary outcomes included the rate of different treatment options (direct to surgery (DTS), non-operative management (NOM), or surgery after trial of NOM (NOM-T)) by hernia type, 30-day in-hospital mortality, complications (graded using the Clavien–Dindo classification), failure-to-rescue (FTR) following complications, and LOS. Secondary outcomes included operative intervention rates and surgery timing^8^ Collected variables encompassed demographic data, co-morbidities, hernia type, clinical presentation, radiological findings (for example, bowel ischaemia, pneumatosis, transition point), management strategies, mesh characteristics (where used; for example, polypropylene, VICRYL™ (Ethicon LLC, Raritan, NJ, USA), biologic), and institutional characteristics (teaching status, surgical resource availability). Data on surgical procedures included rates of small bowel resection and stoma formation. Functional bowel recovery was measured using the PRO-diGI tool across multiple symptom domains^9^.

### Data collection and management

Standardized data collection instruments captured the above variables of interest. Local investigators ensured real-time data entry into a secure, password-protected, GDPR-compliant REDCap^®^ database (version 14.8.3; Vanderbilt University, Nashville, TN, USA), hosted at the University of Pennsylvania. Each site validated >95% completeness of mandatory fields accompanied by quality audits to ensure data integrity.

### Statistical analysis

Descriptive statistics were used to summarize baseline characteristics and management patterns. Continuous variables are presented as the mean with standard deviation (s.d.) or as the median with interquartile range (i.q.r.) depending on data distribution and were compared using two-tailed *t* tests or Mann–Whitney *U* tests, as appropriate. Categorical variables are presented as numbers and frequencies and were compared using χ^2^ or Fisher’s exact tests. Statistical significance was set at *P* < 0.05, with the Benjamini–Hochberg method used to adjust for multiple comparisons. All analyses were conducted using R version 4.1.2 (R Foundation for Statistical Computing, Vienna, Austria) and jamovi version 2.6.2.0 (The jamovi project; https://www.jamovi.org). Figures were generated with BioRender® (https://app.biorender.com).

## Results

### Participants

**Fig. 1:**
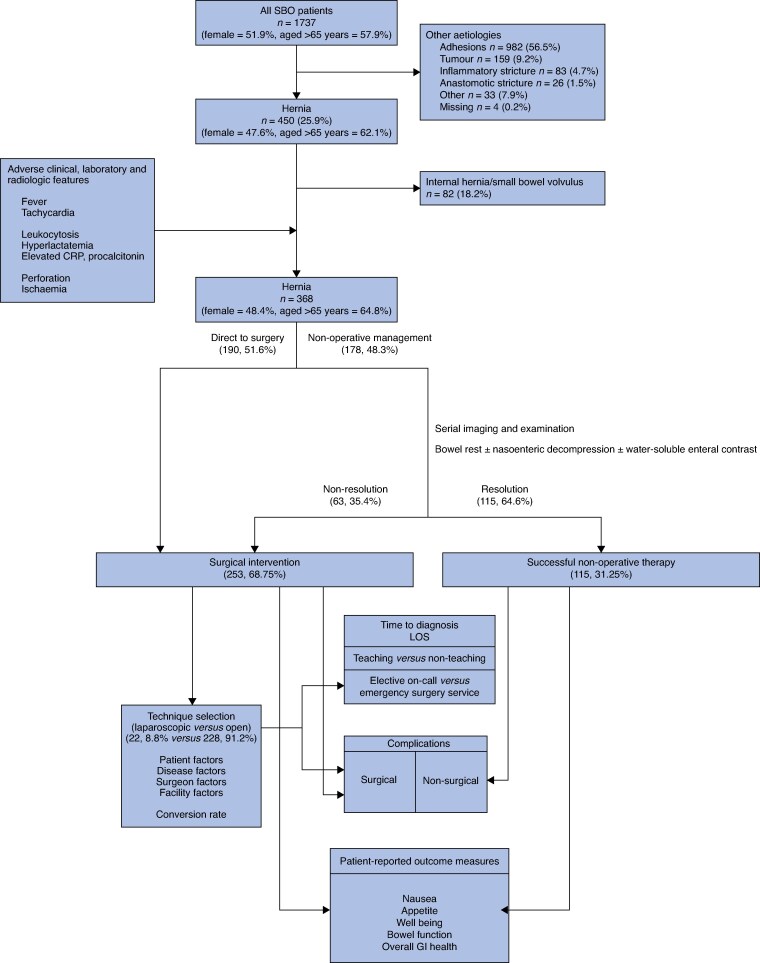
Patient flow diagram SBO, small bowel obstruction; LOS, length of hospital stay; GI, gastrointestinal.

Of the 1737 patients with radiologically confirmed mechanical SBO, 368 (21%) SOBs were attributed to abdominal wall hernias across 70 centres in 20 countries. A flow chart of patient inclusion is shown in *Fig. 1*.

University hospitals cared for 300 of 368 patients (81.5%), with the remaining 18.5% of patients cared for by centres not affiliated with a university. Centres with surgical residents took care of 97.5% of patients. As indicated in *Table 1*, the median patient age was 73 years (i.q.r. 59–83) years, with 64.8% of patients aged > 65 years. Patients were predominantly male (51.6%), with a mean(s.d.) body mass index (BMI) of 28.6(7.43) kg/m^2^. Hernia types were categorized into three groups: primary groin or abdominal wall; incisional; or parastomal. Patients with incomplete data or SBO due to other aetiologies, such as adhesions, luminal obstruction, internal hernia, or malignancy, were excluded. Primary inguinal of abdominal wall hernias were the most common (62.2%; 229 of 368), followed by incisional hernias (28.0%; 103 of 368), and parastomal hernias (9.8%; 36 of 368; *Table 1*). The mean patient BMI was consistent across hernia groups (28.3(7.2) kg/m^2^). The mean(s.d.) LOS was 6.6(8.2) days and did not differ significantly by hernia type (*P* = 0.136).

**Table 1:** Summary of patient characteristics, hernia types, interventions, and outcomes

	Direct to surgery (*n* = 190)	Surgery after trial of NOM (*n* = 63)	Successful NOM (*n* = 115)	Total (*n* = 368)	*P*
**Patient demographics**					
Sex					0.866
Male	100 (52.6%)	33 (52.4%)	57 (49.6%)	190 (51.6%)	
Female	90 (47.4%)	30 (47.6%)	58 (50.4%)	178 (48.4%)	
Age (years), median (i.q.r.)	73.0 (57.0–84.0)	69.0 (59.2–80.5)	75.0 (64.0–83.0)	73.0 (59.0–83.0)	0.315
Age > 65 years	113 (59.5%)	40 (64.5%)	84 (73.7%)	237 (64.8%)	0.043
BMI (kg/m^2^), mean(s.d.)	28.1(7.1)	29.0(7.8)	29.1(7.8)	28.6(7.4)	0.471
Co-morbidities	144 (75.8%)	52 (82.5%)	97 (90.7%)	293 (81.4%)	0.007
**Hospital characteristics**					
Teaching hospital	180 (97.3%)	62 (100.0%)	105 (96.3%)	347 (97.5%)	0.332
Operating room available 24/7	181 (96.8%)	61 (98.4%)	101 (92.7%)	343 (95.8%)	0.124
**Hernia type**					<0.001
Incisional	39 (20.5%)	20 (31.7%)	44 (38.3%)	103 (28.0%)	
Parastomal	7 (3.7%)	7 (11.1%)	22 (19.1%)	36 (9.8%)	
Primary inguinal/abdominal wall	144 (75.8%)	36 (57.1%)	49 (42.6%)	229 (62.2%)	

Values are *n* (%) unless otherwise stated. NOM, non-operative management; i.q.r., interquartile range; BMI, body mass index; s.d., standard deviation.

### Treatment strategies

Three treatment groups were identified: DTS, NOM-T, and successful NOM management. In total, 51.6% of patients (190 of 368) underwent DTS, 17.1% (63 of 368) pursued NOM-T, and 31.2% (115 of 368) successfully completed NOM. The distribution of management strategies differed by hernia type (*P* < 0.001). Specifically, DTS rates were highest for patients with primary inguinal hernias (62.9%; 144 of 229), whereas the highest rate of NOM-T was seen in the group with parastomal hernias (19.4%; 7 of 36). NOM was most common for parastomal hernias (61.1%; 22 of 36) rather than for incisional (42.7%; 44 of 103) or primary inguinal (21.4%; 49 of 229) hernias (*Table 1*). Parsed by intention-to-treat NOM and surgery cohorts, patients who were managed with non-operative strategies were significantly more likely to be aged > 65 years (70.5% *versus* 59.5%; *P* = 0.037) and to have co-morbidities (87.6% *versus* 75.8%; *P* = 0.006); however, median age was identical across groups (73.0 years; *P* = 0.696).

### Surgical technique

Surgery was performed in 253 patients (190 in the DTS group, 63 in the NOM-T group). Most procedures were open (171 of 190 (90.5%) *versus* 57 of 63 (93.4%) for DTS *versus* NOM-T; overall 228 of 253 (91.2%); *P* = 0.652; *Table 2*). Among 221 of 253 hernia repairs (87.4%) for which data regarding surgical technique was available, mesh-mediated repair predominated (using non-biologic mesh, either polypropylene or VICRYL™; 144 of 221, 65.2%), followed by primary suture repair (69 of 221, 31.2%) and biologic prosthetic repair (8 of 221, 3.6%). A biologic prosthesis was predominantly used in the USA (50%) and UK (25%), whereas non-biologic mesh repair was most frequent in Spain (49.3%). Of note, data regarding VICRYL™ bridging mesh, used in six contaminated fields as a temporary onlay, have been presented separately from polypropylene. Suture repair was more commonly performed in Mexico (14.7%) and Serbia (19.1%). In addition, no significant interaction was observed between hernia repair techniques and patient age or co-morbidities. Mean(s.d.) age was similar across the biologic mesh, polypropylene/VICRYL™ mesh, and suture repair groups (58.8(12.9), 70.3(16.2), and 69.8(16.4) years, respectively; *P* = 0.153) Similarly, co-morbidity frequency (82.4% overall prevalence) did not vary between repair approach groups (*P* = 0.399). Among 14 parastomal repairs, one used biologic mesh, five used polypropylene, three used primary suture repair, and five lacked technique data. No differences in bowel resection, anastomosis, or stoma formation rates were noted for DTS *versus* NOM-T.

**Table 2:** Procedural and outcome comparisons between direct to surgery and surgery after a trial of NOM

	Direct to surgery (*n* = 190)	Surgery after trial of NOM (*n* = 63)	Total (*n* = 253)	*P*
Time to surgery (h), mean(s.d.)	10.6(11.7)	30.9(28.5)	15.2 (19.0)	<0.001
Duration of surgery (min), mean(s.d.)	102.5(54.6)	104.5(50.4)	103.0 (53.5)	0.806
Surgery duration > 90 min	90 (47.4%)	34 (54.0%)	124 (49.0%)	0.446
**Surgical approach**				0.652
Laparoscopic	18 (9.5%)	1 (6.6%)	19 (7.7%)	
Open	171 (90.5%)	57 (93.4%)	228 (92.3%)	
Laparoscopic converted to open	7 of 18 (38.9%)	1 of 1(100%)	8 of 19 (42.1%)	
Missing	1	5	6	
Damage control	1 (0.5%)	6 (9.8%)	7 (2.8%)	0.001
**Hernia repair technique**				0.086
Mesh repair (biologic)	5 (3.0%)	3 (5.5%)	8 (3.6%)	
Mesh repair (VICRYL™)	3 (1.8%)	3 (5.5%)	6 (2.7%)	
Mesh repair (polypropylene)	111 (66.9%)	27 (49.1%)	138 (62.4%)	
Sutured tissue repair only	47 (28.3%)	22 (40.0%)	69 (31.2%)	
Missing	24	8	32	
Bowel resection	58 (30.5%)	13 (20.6%)	71 (28.1%)	0.489
Anastomosis	51 of 58 (87.9%)	11 of 13 (84.6%)	62 of 71 (87.3%)	1.000
Stoma formation	7 of 58 (12.1%)	2 of 13 (15.4%)	9 of 71 (12.7%)	0.571
**Intraoperative complications**				
Enterotomy	2 (1.1%)	4 (6.3%)	6 (2.4%)	0.492
Serosal injury	9 (4.7%)	7 (11.1%)	16 (6.3%)	1.000
Haemorrhage	7 (3.7%)	6 (9.5%)	13 (5.1%)	0.774
**Outcomes**				
Length of hospital stay (days), mean(s.d.)	6.4(8.1)	10.2(9.5)	6.6(8.2)	<0.001
Postoperative length of hospital stay (days), mean(s.d.)	6.3(7.9)	7.2(9.4)	6.5(8.3)	0.471

Values are *n* (%) unless otherwise stated. NOM, non-operative management; h, hours; s.d., standard deviation; min, minutes.

### Overall complications

Between-group analysis of overall complications was performed (*Table 3*). In-hospital death occurred in 20 of 368 (5.4%) patients. There was no significant difference in in-hospital mortality between the DTS (3.8%) and NOM (5.1%) groups (*P* = 0.560), but was significantly higher in the NOM-T group (9.2%; *P* < 0.001).

**Table 3:** Comparison of surgical and non-operative complications and their severity between groups, according to treatment received

	Direct to surgery	Surgery after trial of NOM	Successful NOM	Total	*P*
No. of patients (%)	190 (51.6%)	63 (17.1%)	115 (31.2%)	368 (100%)	
**Clavien–Dindo classification of complications**					< 0.001
No complications	129 (67.9%)	40 (63.5%)	107 (93.0%)	276 (75.0%)	
Grade I: Any deviation from the normal postoperative course not requiring surgical, endoscopic or radiological intervention; this includes the need for certain drugs (for example anti-emetics, antipyretics, analgesics, diuretics, and electrolytes), and wound infections that are opened at the bedside	21 (11.1%)	4 (6.3%)	1 (0.9%)	26 (7.1%)	
Grade II: Complications requiring drug treatments other than those allowed for Grade I complications; this includes blood transfusion and TPN	18 (9.5%)	7 (11.1%)	2 (1.7%)	27 (7.3%)	
Grade IIIa: Surgical, endoscopic or radiological intervention not under general anaesthetic	4 (2.1%)	0 (0.0%)	0 (0.0%)	4 (1.1%)	
Grade IIIb: Surgical, endoscopic or radiological intervention requiring general anaesthetic	5 (2.6%)	5 (7.9%)	0 (0.0%)	10 (2.7%)	
Grade IVa: Life-threatening single-organ dysfunction; this includes CNS complications (for example brain haemorrhage, ischaemic stroke, subarachnoid haemorrhage) that require intensive care (including dialysis)	4 (2.1%)	1 (1.6%)	0 (0.0%)	5 (1.4%)	
Death	9 (4.7%)	6 (9.5%)	5 (4.3%)	20 (5.4%)	
Any grade of complication	61 (32.1%)	23 (36.5%)	8 (7.0%)	92 (25%)	
**Medical complications**					
Aspiration pneumonia	9 (4.7%)	3 (4.8%)	1 (0.9%)	13 (3.5%)	0.175
Deep venous thrombosis	2 (6.1%)	1 (9.1%)	0 (0.0%)	3 (6.0%)	0.752
Pulmonary embolism	2 (6.5%)	1 (9.1%)	1 (16.7%)	4 (8.3%)	0.706
Myocardial infarction	1 (3.1%)	0 (0.0%)	0 (0.0%)	1 (2.0%)	0.762
Cerebrovascular accident	1 (3.1%)	0 (0.0%)	0 (0.0%)	1 (2.0%)	0.762
Acute renal failure	7 (21.9%)	8 (72.7%)	5 (83.3%)	38 (77.6%)	0.874
Respiratory failure	10 (31.2%)	4 (36.4%)	3 (42.9%)	17 (34.0%)	0.436
Postoperative delirium	4 (12.9%)	0 (0.0%)	0 (0.0%)	4 (8.3%)	0.302
Cardiac arrest	8 (25.8%)	2 (18.2%)	5 (71.4%)	15 (30.6%)	0.036

Values are *n* (%). NOM, non-operative management; TPN, total parenteral nutrition; CNS, central nervous system; TIA, transient ischaemic attacks.

Based on the Clavien–Dindo classification, 276 of 368 patients (75%) experienced no complications (Grade 0), whereas 92 patients experienced at least one complication: 61 (32.1%) in the DTS group, 23 (36.5%) in the NOM-T group, and 8 (7.0%) in the NOM group. Overall, 53 patients (14.4%) required non-operative treatment for surgical complications (Grade I–II), 14 (3.8%) required operative or radiological intervention (Grade IIIa or IIIb), and 5 (1.4%) required admission to a critical care setting for repair of organ failure (Grade IV).

Non-operative complications were observed in 18.8% (69 of 368) of patients. Surgical complications occurred in 22.4% (57 of 253) of patients treated with DTS and NOM-T, with a significantly (*P* = 0.005) higher incidence in the parastomal hernia group (57.1%; 8 of 14) than in the incisional (23.7%; 14 of 59) or primary inguinal (19.3%; 35 of 181) hernia groups (*Table 4*). Surgical site infections represented 48.1% of postoperative complications, and postoperative haemorrhage occurred in 7.1% of patients. However, the mean(s.d.) postoperative LOS was significantly longer in the parastomal hernia group than in the incisional and primary inguinal hernia groups (9.1(4.8) *versus* 8.7(10.5) and 5.6(7.4) days, respectively; *P* = 0.030). Among the 92 patients who developed complications, the FTR rate differed significantly between the DTS, NOM-T, and NOM groups: 14.8% (9 of 61), 26.1% (6 of 23), and 62.5% (5 of 8), respectively (*P* < 0.001).

**Table 4:** Patient and procedural characteristics according to hernia type

	Hernia type			Total (*n =* 368)	*P*
	Incisional (*n =* 103)	Parastomal (*n =* 36)	Primary inguinal/abdominal wall (*n =* 229)		
**Patient demographics**					
Sex					0.001
Male	37 (35.9%)	20 (55.6%)	133 (58.1%)	190 (51.6%)	
Female	66 (64.1%)	16 (44.4%)	96 (41.9%)	178 (48.4%)	
Age (years), median (i.q.r.)	69.0 (56.0–79.0)	76.5 (67.5–83.8)	75.0 (59.0–84.0)	73.0 (59.0–83.0)	0.007
Age ≥ 65 years	61 (59.2%)	28 (82.4%)	148 (64.6%)	237 (64.8%)	0.050
BMI (kg/m^2^), mean(s.d.)	31.1(8.3)	28.0(6.3)	27.5(6.9)	28.6(7.4)	<0.001
**Length of hospital stay (days), mean(s.d.)**					
Total	7.7(10.2)	8.2(7.8)	5.9(7.2)	6.6(8.2)	0.136
Postoperative	8.7(10.5)	9.1(4.8)	5.6(7.4)	6.5(8.3)	0.030
**Intervention cohort**					<0.001
Direct to surgery	39 (37.9%)	7 (19.4%)	144 (62.9%)	190 (51.6%)	
Surgery after trial of NOM	20 (19.4%)	7 (19.4%)	36 (15.7%)	63 (17.1%)	
Successful NOM	44 (42.7%)	22 (61.1%)	49 (21.4%)	115 (31.2%)	
**Perioperative non-surgical interventions**					
NG placed	65 (63.1%%)	26 (72.2%%)	143 (62.4%)	234 (63.6%)	0.462
Water-soluble enteral contrast challenge	21 (20.4%)	11 (30.6%%)%)	12 (5.2%)	44 (12.0%)	<0.001
Surgical technique					
**Patients with hSBO undergoing surgery**	59 (57.3%)	14 (38.9%)	180 (78.6%)	253(68.8%)	<0.001
Sutured tissue repair only	19 (37.3%)	3 (33.3%)	47 (29.2%)	69 (31.2%)	
Mesh repair (biologic)	3 (5.9%)	1 (11.1%)	4 (2.5%)	8 (3.6%)	
Mesh repair (polypropylene)	23 (45.1%)	5 (55.6%)	110 (68.3%)	138 (62.4%)	
Mesh repair (VICRYL™)	6 (11.8%)	0 (0.0%)	0 (0.0%)	6 (2.7%)	
Missing	8	5	19	32 (14.4%)	
**Intraoperative complications**					
Bowel resection	18 (34.6%)	7 (50.0%)	46 (25.6%)	71 (25.8%)	0.036
Stoma formation	3 (5.1%)	2 (14.314.3%)	5 (2.8%)	10 (3.6%)	0.652
Enterotomy repair	5 (8.5%)	1 (7.1%)	0 (0.0%)	6 (2.2%)	0.238
Serosal injury repair	10 (16.9%)	2 (14.3%)	4 (2.2%)	16 (5.8%)	0.344
Haemorrhage	9 (15.3%)	1 (7.1%)	3 (1.7%)	13 (4.7%)	0.215
**Postoperative surgical complications**					
Patients with hSBO submitted to surgery	59 (57.3%)	14 (38.9%)	180 (78.6%)	253(68.8%)	
Surgical reintervention	3 (5.1%)	3 (21.4%)	12 (6.7%)	18 (6.5%)	0.633
Anastomotic leak	0 (0.0%)	0 (0.0%)	4 (2.2%)	4 (1.5%)	0.259
Superficial SSI	9 (15.3%)	4 (28.6%)	18 (10.0%)	31 (12.3%)	0.090
Deep SSI	0 (0.0%)	1 (7.1%)	2 (1.1%)	3 (1.2%)	0.084
Organ space SSI	2 (3.4%)	1 (7.1%)	4 (2.2%)	7 (2.8%)	0.527
Fascial dehiscence	0 (0.0%)	1 (7.1%)	2 (1.1%)	3 (1.1%)	0.442
Postoperative haemorrhage	3 (5.1%)	1 (7.1%)	6 (3.3%)	10 (3.6%)	0.865
Postoperative SBO	0 (0.0%)	2 (14.3%)	7 (3.9%)	9 (3.3%)	0.165

Values are *n* (%) unless otherwise stated. i.q.r., interquartile range; BMI, body mass index; s.d., standard deviation; NOM, non-operative management; NG, nasogastric tube; hSBO, hernia-related small bowel obstruction; SSI, surgical site infection; SBO, small bowel obstruction.

Of the 368 patients included in this study, 251 (68.2%) had a scheduled post-discharge clinical encounter (210 of 253 (83.0%) postoperative patients *versus* 41 of 115 (35.6%) NOM patients; *P* < 0.001), 190 (75.7%) of which were in person in an outpatient clinic and 61 (24.3%) were virtual encounters via telephone or video conferencing software.

### Functional recovery

Patient-reported outcome measures (PROMs) of functional recovery were captured for 218 patients during post-discharge visits using PRO-diGI, with significant differences found according to hernia type. Patients with parastomal hernias had lower overall bowel function scores (mean(s.d.) 68.6(22.5)) than those with primary inguinal (82.0(20.3)) or incisional (83.6(17.6)) hernias (*P* = 0.009). Bowel-specific recovery was also significantly worse in the group with parastomal hernias than in the groups with primary inguinal and incisional (mean(s.d.) scores 78.6(19.6) *versus* 91.5(16.4), and 90.6(15.7), respectively; *P* = 0.005). Other functional metrics, including appetite (*P* = 0.473), nausea (*P* = 0.855), and general well being (*P* = 0.455), did not differ significantly according to hernia type.

## Discussion

Within the broader spectrum of intestinal obstruction, hSBO is a distinct clinical entity^10^. In the present multicentre time-bound prospective observational study cohort, hSBO ranked as the second most frequent cause of obstruction after adhesive SBO, mirroring patterns observed in healthcare systems in high-income countries^11^. These systems generally have robust diagnostic capabilities and experienced surgical teams, which can influence both the speed and type of intervention provided. There is a knowledge gap in the literature, with a targeted literature search showing that very few studies have assembled cohorts approaching the size of the data set used in the present study spanning 6 months and including 368 patients. The only clearly comparable prospective series is the UK National Audit of Small-Bowel Obstruction (NASBO), which enrolled 2341 SBO admissions across 131 hospitals and reported a hernia-specific subgroup of 415 patients, albeit confined to a single healthcare setting in one country^3^. All other published studies are single-centre or narrowly focused case series with fewer than 150 hernia-related cases.

Primary inguinal and abdominal wall hernias emerged as the most prevalent causes within the hSBO category. Incisional and parastomal hernias followed in frequency, matching previously published epidemiological data^3^. Notably, parastomal hernias were found to have especially high complication rates and persistently poor postoperative functional outcomes^12–14^. This finding underscores the need for more detailed research into patient-level characteristics and management strategies tailored to the complexities of hSBO, which includes a spectrum of clinical presentations, stoma-related challenges, and varying degrees of abdominal wall compromise^15–18^. The recent MASH prospective cohort documented marked heterogeneity in mesh-placement planes during emergency hernia repair^2^. Current guidelines offer limited guidance for such acute settings, highlighting a knowledge gap that warrants targeted investigation^2^. Despite well documented guidelines^19,20^ recommending urgent surgery for hSBO to prevent strangulation and bowel ischaemia, a considerable proportion of patients in the present study underwent NOM based on an intention-to-treat model. This included both successful NOM and patients who ultimately required surgical rescue (NOM-T). Advanced imaging technologies and greater access to critical care therapies available in resource-replete institutions may offer clinicians confidence in postponing surgical intervention, especially for patients with borderline physiological profiles. These findings suggest that institutional protocols, local resources, and the presence of integrated acute care surgery teams can all contribute to practice variation.

Each major hernia subtype encountered presents differences in pathophysiology, surgical history, and anatomical challenges, and demands a distinct clinical approach. Primary inguinal and abdominal wall hernias are often considered less complex to repair because they commonly present without the complicating factors of previous mesh or dense adhesions. However, incisional hernias may involve extensive scar tissue, uncertain bowel positioning from previous procedures, and varying degrees of mesh incorporation into the abdominal wall. These factors can obscure the exact cause of obstruction, leading to caution or NOM until the surgical team is confident that the incisional hernia is, indeed, the precipitating factor^20,21^. Parastomal hernias remain the most challenging subset because they occur in the setting of a stoma, which alters normal abdominal wall anatomy and increases the risk of infection, strangulation, and complicated surgical repairs^14,15,22^. Guidelines that address parastomal hernia repair strategies typically focus on elective scenarios^13^. Thus, emergency parastomal hernia care, often hinging on individual-surgeon judgement informed by experience, exhibits variable patient outcomes, which highlights the need for more evidence-based prescriptive advice.

The decision to pursue operative repair in hSBO depends largely on an assessment of the anticipated likelihood of success. Local resource availability, including operating room capacity, staffing, and scheduling constraints, can significantly shape the initial clinical pathway for hSBO, whereas patient factors such as a high BMI, advanced age, extensive co-morbidities, and the use of certain pharmacological agents can diminish wound healing and increase the risk of perioperative complications^23^. Surgeons may therefore choose a period of non-operative management (NOM or NOM-T) to correct metabolic derangements or coordinate multidisciplinary input. Similarly, in certain lower-resource settings, operating room availability and the cost or availability of mesh materials can limit the feasibility of immediate repair^24^. In this context, the decision to proceed with operative management becomes more complex, shaped by both clinical and logistical considerations.

In tertiary or quaternary referral centres managing multiple urgent conditions, patients with hSBO may be triaged behind those with more immediate life-threatening issues. Even though these institutions have sophisticated equipment and highly trained personnel, the sheer volume of emergency operations can limit the feasibility of prompt surgery for every patient with SBO. As a result, surgeons may choose to stabilize patients overnight in an initially non-operative treatment pathway (NOM-T) before planned surgery the next day^25–28^. Although this approach can reduce perioperative risks by allowing targeted fluid resuscitation and repair of electrolyte imbalances, it may also extend the hospital stay and add complexity to the patient’s clinical course. This pathway emphasizes the importance of well structured triage guidelines that balance emergency needs with the potential benefits of optimization.

In the present cohort, complications in the parastomal hernia group reached 57.1% (for a total number of 36), which exceeds the complication rates for incisional (23.7%) and primary inguinal hernias (19.3%). Surgical site infections were the primary source of morbidity in patients with parastomal hernias, likely reflecting the interaction between altered local anatomy and the stoma’s impact on skin integrity. These challenges translated into notably worse patient-reported functional recovery, as indicated by significantly (*P* = 0.009) lower mean(s.d.) bowel function scores (68.6(22.5)) than for the incisional (83.6(17.6)) and primary inguinal (82.0(20.3)) hernia groups. Although the findings of the present study are encouraging, it is essential to acknowledge that the conclusions are derived from a very limited patient cohort. Future research specifically tailored to hSBO is warranted to generate more robust and generalizable evidence for this clinical context. Although traditional clinical metrics such as mortality, LOS, and readmission rates retain their utility, PROMs enrich the holistic understanding of how complications affect daily living, mental well being, and social functioning. Integrating PROMs into routine post-hospitalization follow-up would support cross-institutional benchmarking, encourage the development of patient-centred quality improvement initiatives, and potentially aid in tailoring postoperative interventions to specific hernia types, an approach that aligns with the European Society for Trauma and Emergency Surgery’s research agenda^29–31^.

Among the patients in this study who developed complications, the FTR differed significantly between groups. Although these data may, at first reading, suggest a causal relationship between non-operative treatment and mortality once complications arise, patient selection is likely driving much of this effect. Because surgeons frequently reserve NOM for those with compromised physiology, extensive co-morbidities, and in the absence of signs of intestinal ischaemia or perforation, the higher FTR in the NOM cohort may reflect the co-morbidity burden rather than the management strategy itself. Conversely, DTS patients are generally younger, with fewer co-morbidities, and present more compelling indications for operative intervention, leading to a lower observed FTR. Future research using advanced causal inference techniques, including but not limited to the use of propensity scores and comprehensive risk stratification, could further disentangle whether FTR differentials primarily result from patient factors or from the timing and nature of the intervention. Preoperative risk assessment tools are designed to bring objectivity and data-informed perspectives to surgical decision-making. The Predictive Optimal Trees in Emergency Surgery Risk (POTTER) calculator^32^, the Portsmouth Physiological and Operative Severity Score for the Enumeration of Mortality and Morbidity (P-POSSUM)^33^, and the NSQIP surgical risk calculator^34^ all serve to stratify perioperative risk in various patient populations. When combined with instruments that measure sepsis, organ dysfunction, and frailty, these tools can offer a more holistic view of a patient’s status and expected trajectory^35–37^. Structured evaluation incorporating the prognostic value of these validated tools can then guide dialogue between clinicians, patients, and their families about the benefits and risks of surgery *versus* continued NOM.

This multicentre snapshot audit provides broad insights into real-world practice but is subject to several limitations. First, variations in data completeness across institutions may bias the results, and key variables, such as specific repair materials, surgical methods, and long-term outcomes, remain insufficiently detailed. Second, the study’s focus on high-resource healthcare systems limits extrapolation to low- and middle-income contexts, where hSBO may present differently. Third, the observational nature of the study design restricts causal inferences about operative timing and repair techniques on clinical and functional endpoints. Data on the diameter of the hernia defect were not collected; future audits should capture this variable because size may influence surgical decision-making. The operative case report form did not capture the anatomical plane of mesh implantation (onlay, sublay, retromuscular, or intraperitoneal). Mesh position influences mechanical load, contamination risk, and subsequent recurrence, and guides the selection of reinforcement at any future repair^38^. Omission of this variable prevents stratified outcome analysis and limits comparability with European Hernia Society recommendations^13^ that mandate plane-specific reporting in emergency hernia audits^39^. Fourth, the lack of standardized post-discharge follow-up protocols and limited collection of PROM data impede a thorough assessment of long-term recovery. Finally, this study is a subgroup analysis of a larger study designed for all types of SBOs; the non-specific design of the study may introduce inclusion and exclusion criteria bias and restrict the number of overall patients, leading to inconsistent conclusions. Patients with a parastomal hernia constituted a small subgroup of the study; the related findings should be regarded as hypothesis-generating until confirmed in larger studies. These constraints highlight the need for prospective studies using harmonized data collection across diverse healthcare environments.

In conclusion, hSBO management exhibits substantial variability, driven by hernia aetiology and clinical decision-making paradigms. SnapSBO identified patients with parastomal hernias as a high-risk subgroup, with significantly higher complication rates, prolonged hospital stays, and worse functional recovery metrics. The findings underscore the need for refined risk stratification tools to guide patient selection for early surgical intervention, particularly where delayed operative management may exacerbate morbidity. Current practice patterns reflect an absence of standardized evidence-based protocols, leading to disparate outcomes across institutions. The observed high rates of NOM failure in patients with a parastomal hernia suggest knowledge gaps in decision-making. Integrating PROMs into routine care offers holistic recovery evaluation beyond traditional surgical endpoints. Future evidence-informed guideline development should prioritize accessible aetiology-specific management pathways that balance the risks and benefits of surgical intervention and improve global consistency in hSBO care.

## Collaborators

Members of the ESTES SnapSBO Steering Group: Matteo Maria Cimino (Fondazione IRCCS Ca’ Granda Ospedale Maggiore Policlinico, Milan, Italy); Gary Alan Bass (University of Pennsylvania, Philadelphia, PA, USA); Isidro Martinez-Casas (Hospital Universitario Virgen del Rocio in Sevilla, Spain); Lewis J. Kaplan (University of Pennsylvania, Philadelphia, PA, USA); Hayato Kurihara (Fondazione IRCCS Ca’ Granda Ospedale Maggiore Policlinico, Milan, Italy); Matthew J. Lee (University of Birmingham, UK); Shahin Mohseni (Orebro University, Sweden). Members of the ESTES SnapSBO Group: Matteo Porta (Fondazione IRCCS Ca’ Granda Ospedale Maggiore Policlinico, Milan, Italy); Pavel Karasek (University of Pennsylvania, Philadelphia, PA, USA); Agron Dogjani, Kastriot Subashi, Klevis Doci, Joana Spaho (University Hospital of Trauma, Tirana, Albania); Abdulla, Sara Ahmed, Yusuf AlAnsari, Mariam AlKooheji, Alaa Marzooq, Khaled Nazzal (Salmaniya Medical Complex, Bahrain, UAE); Emir Ahmetašević, Zlatan Mehmedović, Maja Kovačević, Jasminka Mujkanović (University Clinical Centre Tuzla, Bosnia and Herzegovina); Peter Svenningsen, Marie Peter Møller, Gitte Emilje Olsen (Nordsjællands Hospital - University of Copenhagen, Denmark); Abeer Aboalazayem, Muhammad Ashrad Awad, Mahmoud M. A. Elfiky, Moemen Farouk, Mostafa Gad, Basma Magdy (Cairo University, Egypt); Peep Talving, Edgar Lipping, Edgar Lipping, Sten Saar, Artjom Bahhir, Maarja Talviste (North Estonia Medical Centre, Talinn, Estonia); Vincent Dubuisson, Luca Cigagna (Centre Hospitalier Universitaire de Bordeaux, France); Spyridon Christodoulou, Panagiotis Kokoropoulos, Ioannis Margaris, Maria Papadoliopoulou, Theodoros A. Sidiropoulos, Panteleimon Vassiliu (Attikon University Hospital, Greece); Evangelos Barkolias, Pavlos Georgalis, Theodosios Kantas, Vasiliki Nikolaou, Aristeidis Papadopoulos, Katerina Tata (General Hospital of Nikaia, Greece); Stergios Arapoglou, Ioannis Gkoutziotis, Aikaterini Mpratko, Elissavet Symeonidou (Ippokrateio General Hospital, Greece); Stylianos Kykalos, Nikolaos Machairas, Adam Mylonakis, Panagiotis Sakarellos, Dimitrios Schizas, Michail Vailas (Laikon General Hospital, Greece); Iraklis Anastasiadis, Parmenion Patias, Koumarelas Konstantinos, Mourtarakos Saradis (Nafplio General Hospital, Greece); Charles Lee, Chloe Spillane, Dylan Viani Walsh, Nadia Walsh, Arnold D. K. Hill, Thomas Noel Walsh (Beaumont Hospital Dublin, Ireland); Gabriel Orsi, Andrew Keane, David Kearney, Emma de Sousa (Connolly Hospital Dublin, Ireland); Michael Sugrue, Anne Marie Doyle, Robert Fitzsimmons, Angus J. Lloyd, Mohammad Saad Qasim, Mashood Ahmed (Letterkenny University Hospital, Ireland); Taylor Jacoby, Michael E. Kelly, Shafagh Khodadi, Paul McCormick, Éanna J. Ryan, Mahmoud M. Salama (St. James University Hospital, Dublin, Ireland); Helen Heneghan, Cian Davis, Odhran K. Ryan, Sean T. Martin (St. Vincent’s University Hospital, Dublin, Ireland); Miklosh Bala (Hadassah Medical Center and Faculty of Medicine, Hebrew University of Jerusalem, Israel); Michele Altomare, Stefano P. B. Cioffi, Andrea Spota, Giada Panagini, Laura Benuzzi, Stefania Cimbanassi (ASST Grande Ospedale Metropolitano Niguarda, Italy); Noemi DiFuccia, Stefano Manfroni (Azienda Ospedaliera San Camillo Forlaninin, Italy); Alan Biloslavo, Paola Germani, Nicolo de Manzini, Manuela Mastronardi, Anna Modica, Serena Scomersi (Cattinara University Hospital, Trieste, Italy); Gabriele Bellio, Luigi Cayre, Gaia Altieri, Pietro Fransvea, Gabriele Sganga, Silvia Tedesco (Fondazione IRCCS Ca’ Granda Ospedale Maggiore Policlinico, Milan, Italy); Francesca Bunino, Sabrina Caspani, Daniele DelFabbro, Simone Giudici, Giulia Mauri, Paolo Meneghesso (IRCCS Humanitas Research Hospital, Milan, ITaly); Enrico Ortolano, Carolina Perali, Antonella D'addiego, Francesca Di Vittorio, Gabriele Bormolini, Michele Carlucci (IRCCS Ospedale San Raffaele, Italy); Alessia Malagnino, Maria Ilaria Ficaccio, Giovanni Pesenti, Claudia Tintori, Mauro Zago (Ospedale Alesandro Manzoni, Italy); Martina Zambon, Simona Meneghini, Andrea Mingoli, Giulia Duranti, Gioia Brachini, Pierfrancesco Lapolla, Mehdi Hanafi (Policlinico Umberto I, Italy); Clara Valdez Cruz, Andrea Alfredo Huerta de León, Jose García Regalado, Pasquale de Jesús Cristiano Nakhal, Diego Enrique Rodríguez González (Nuevo Hospital Civil de Guadalajara “Dr. Juan I. Menchaca”, Mexico); Jose Alaniz-Ruiz, Edgard Lozada-Hernández, Salvador Carlos-Jiménez, Oscar Reyes-Delgado, Monserrat Martínez-Zamorano (Hospital Regional de Alta Especialidad del Bajío, Mexico); Ademola Adetoyese Adeyeye, Ehis Afeikhena, Akinola Akinmade, Babatunde Mustapha (Afe Babalola University Multisystem Hospital, Nigeria); Jaroslav Presl, Patrick Rebnegger, Bjoern Rudisch, Gruenfelder Johanna, Rokitte Karin (Paracelsus Medical University, Austria); Filipa M. CorteReal, Jorge A. Pereira, Joao L. Pinheiro, Daniela M. Pinto, Andreia J. Santos, Andreia M. Silva (Centro Hospitalar Tondela, Portugal); Susana Henriques, Joao Melo, António Miguel Pereira, Antonio Miguel Pereira (Hospital Garcia de Orta, Portugal); Ana Margarida Cabral, Bruno Dias Couto, Barbara Nunes Gama, Catarina Santos Rodrigues (Hospital da Horta, EPER, Portugal); Mara Nunes, Bruno Ribeiro Silva, Daniela Tavares (Unidade Local de Saúde de Matosinhos - Hospital Pedro Hispano, Portugal); Toma Mihai, Oprea C. Valentin (‘Constantin Papilian’ Emergency Clinical Military Hospital of Cluj-Napoca, Romania); Srdjan S. Putnik (General Hospital Vršac, Serbia); Petar Andjic, Marija Djujic, Rastislav Filko, Vanja Kunkin, Andjela Milak, Aleksandar Ognjenovic (General Hospital Đorđe Joanović, Serbia); Nebojša Mitrović, Dejan Stevanović, Damir Jašarović, Goran Aleksandrić, Nemanja Trifunović (Zemun Hospital, Serbia); Mihailo Bezmarević, Sasa Dragović, Milan Jovanović, Bosko Milev, Miroslav Mitrović, Srdjan Petković (Serbian Military Academy, Serbia); Valentina Isakovic, Nikola Zoran Nikolic, Predrag Radic, Dragan Luka Vasic (Hospital Novi Sad, Serbia); Zlatibor M. Loncar, Dusan D. Micic, Vladimir R. Resanovic, Pavle D. Vladimir; Krstina S. Doklestic Vasiljev, Zlatibor M. Loncar, Dusan D. Micic, Vladimir R. Resanovic, Pavle D. Gregoric (Clinical Center of Serbia; Medical Faculty, University of Belgrade, Serbia); Ljiljana Velibor Milic, Vladica Velibor Cuk, Jovan Todor Juloski, Radisav Slavoljub Radulovic, Dragana Dragan Arbutina (University Clinical Hospital Center “Zvezdara” Clinic for Surgery “Nikola Spasic”, Serbia); Jacobo Trebol, Isaac Tapiador-López, Manuel J. Torres-Jurado, Andres E. Valera-Montiel, Francisco Blanco-Antona (Complejo Asistencial Universitario de Salamanca, Spain); Beatriz de Andrés-Asenjo, Maria Ruiz-Soriano, Tania Gómez-Sanz, Andrea Vázquez-Fernández, Juan Beltran de Heredia (Hospital Clínico Universitario de Valladolid, Spain); Cristina Rey-Valcárcel, Monica Ballón-Bordon, Maria Pérez-Díaz, Maria Dolores Sanchez-Rodriguez, Jose David Gonzalez-Esteban (Hospital General Universitario Gregorio Marañón, Spain); Celia Alegre Nevado, Ricardo Montenegro Romero (Hospital Nuestra Señora de Sonsoles, Spain); Inés Capitán del Río (Hospital San Juan de Dios del Aljarafe, Spain); Andrea Campos-Serra, Raquel Gracia-Roman, Heura Llaquet-Bayo, Anna Muñoz-Campaña, Giulia Vitiello (Hospital Universitari Parc Taulí, Spain); Lorena Apodaca Murguiondo, Inigo Augusto Ponce, Amaia Garcia Dominguez, Aintzane Lizarazu Perez (Hospital Universitario Donostia, Spain); Elena Sagarra Cebolla, Mónica García Aparicio, Paloma Garaulet González, Benito Miguel Josa Martínez, Miriam Fraile Vasallo (Hospital Universitario Infanta Cristina, Spain); Mónica Mengual Ballester, Isabel Andrés Lucas Zamorano, Jose Martinez Moreno, Manuel Luis Buitrago Ruiz, Clara Piñera Morcillo (Hospital Universitario J. M Morales Meseguer, Spain); Alberto Díaz García, Hanna Hernández Oaknin, Maria Pellicer Barreda, Jennifer Amparo García Niebla, Antonio Pérez Álvarez (Universitario Nuestra Señora de Candelaria, Spain); Diego Cordova, Laura Jiménez, Fernando Mendoza, Cristina Vera, Alberto Vilar Tabanera (Hospital Universitario Príncipe de Asturias, Spain); María de los Ángeles Gil-Olarte Márquez, José Antonio López-Ruiz, Mª Estela Romero-Vargas, Julio Reguera-Rosal, Alberto García-García, Beatriz Marenco de la Cuadra (Hospital Universitario Virgen Macarena, Spain); Eduardo Perea del Pozo, Virginia Duran Muñoz, Felipe Pareja Ciuró (Hospital Universitario Virgen del Rocio, Spain); Ainoa Benavides dos Santos, Ernest Bombuy, Anna G-Monferrer, Sandra López Gordo (Hospital de Mataró, Spain); José Guerra, Vanessa Sojo, Begona De Soto, Aaron Roman (Hospital de la Merced, Spain); Ana María González-Castillo, Elena Manzo, Estela Membrilla-Fernandez, Amalia Pelegrina-Manzano, Simone Cremona (Hospital del Mar, Barcelona, Spain); Alexander Forero-Torres, Santiago Valderrabano, Francisco Reinoso Olmedo, Fuad Lopez Fernandez (La Paz University Hospital, Spain); Aitor Landaluce-Olavarria, Jon Barrutia- Leonardo, Alba Garcia-Trancho, Melania Gonzalez-De Miguel, Izaskun Markinez-Gordobil (Urduliz Hospital, Spain); Maryam Makki, Dana Altamimi, Sadhika Vinod (Sheikh Shakhbout Medical City, Abu Dhabi, UAE); Olga Rutka, John V. Taylor (Liverpool University Hospitals NHS Foundation Trust, UK); M. Denton, S. Gourgiotis, R. Ravi, A. J. Ribbits (Addenbrooke's Cambridge University Hospital, UK); Jared Wohlgemut, Niroshini R. Hemadasa, Shehryar Rangana Khan, Christopher Leiberman, Sabreen P. Elbakri, Charlie A. Edgar (University Hospital Wishaw, UK); Conor Magee, Kamalesh Inteti, Oluwaseun Oyekan, Mehwish Ansar, Jeremy Wilson, Rahel Rashid (Wirral University Teaching Hospitals NHS Foundation Trust, UK); Deborah Atwell, Joshua Cassedy, Brianna Gabriel, William Hoff, Shyam Murali (Grand View Health, PA, USA); Anna E. Garcia Whitlock, Therese Murphy, Carolyn Susman, Sarah Barnett, Emily Ertmann, Camden DeSanctis, Pavel Karasek, Nathan Klingensmith (University of Pennsylvania, USA); Dale F. Butler, Brandon Bruns, Ankeeta Mehta, Vanessa Nomellini, Keyur Patel, Anthony Tannous (University of Texas Southwestern Medical Center, USA).

## Data Availability

The data sets generated and analysed during this study are available from the corresponding author, on behalf of the SnapSBO study steering group, upon reasonable request.
